# Cerebrospinal Fluid Leak Presenting as Headache: A Case Report

**DOI:** 10.7759/cureus.64683

**Published:** 2024-07-16

**Authors:** Mark Colantonio, Christopher Dionne

**Affiliations:** 1 Internal Medicine, West Virginia University School of Medicine, Morgantown, USA

**Keywords:** lumbar puncture (lp), lumbar puncture, sphenoid sinus, headache, cerebrospinal fluid leak, cerebrospinal fluid

## Abstract

Headache is a common chief complaint among patients. When presented with this chief complaint, clinicians often form a differential diagnosis of common etiologies, including dehydration, increased stressors, and medication side effects. However, a skillful clinician must always be vigilant of rare etiologies presenting with common chief complaints. Here, we present a rare case of a cerebrospinal fluid leak in a young female presenting with primary symptoms of headache, neck stiffness, and vision changes.

## Introduction

Cerebrospinal fluid (CSF) is a clear, colorless fluid naturally produced by the chordal plexus and found in the subarachnoid space [1]. Although responsible for many vital bodily functions, its composition is relatively simple, including water, electrolytes, glucose, protein, and neurotransmitters [1]. Functions of CSF include acting as an insulator and cushion for the central nervous system (CNS) when exposed to external forces, acting as a lymphatic system to drain toxins and metabolites, and delivering essential nutrients needed to perform vital bodily functions [2,3]. Most importantly, CSF is responsible for maintaining a homeostatic environment of the CNS through continuous recirculation [1]. After production by the chordal plexus, CSF is then re-absorbed by the arachnoid villi [1].

Typically housed in the subarachnoid space, a CSF leak can occur when CSF escapes into the extradural space [1]. Ninety percent (90%) of CSF leaks are secondary to craniofacial trauma to the skull base presenting with rhinorrhea, whereas orbital and temporal bone injuries are less likely to lead to CSF leaks [1,4]. The onset of a CSF leak secondary to trauma varies, with more than half presenting within the first two days and all within three months of trauma [4]. Other etiologies of CSF leaks include iatrogenic causes secondary to endoscopic sinus surgery [1]. In such cases, a damaged ethmoid bone is the most common etiology, followed by a damaged cribriform plate [1]. Diagnostic tests, including lumbar punctures, may also lead to CSF leaks [1]. Much less frequently, spontaneous CSF may occur in roughly 5 out of 100,000 people per year [5]. Common causes of high-pressure leaks presenting with CSF rhinorrhea include tumors and hydrocephalus, whereas normal pressure leaks can be secondary to erosion or congenital anomalies [6]. Most commonly, idiopathic intracranial hypertension (ICH) accounts for 70% of spontaneous CSF leaks and is thought to be secondary to impaired CSF resorption [7]. Obstructive sleep apnea (OSA) is the second most common cause of spontaneous CSF leaks [8]. Intracranial tumors, congenital cranial defects, and idiopathic causes have also been documented [9-11]. Regardless of etiology, 92% of cases present with headache as the primary complaint, followed by nausea and associated neck pain [1]. As noted above, spontaneous CSF leaks are a relatively rare occurrence, with some studies citing a prevalence of 1 in 50,000 individuals. Here, we present a case of a spontaneous CSF leak in a young female presenting with primary symptoms of headache and neck stiffness.

## Case presentation

**Figure 1:**
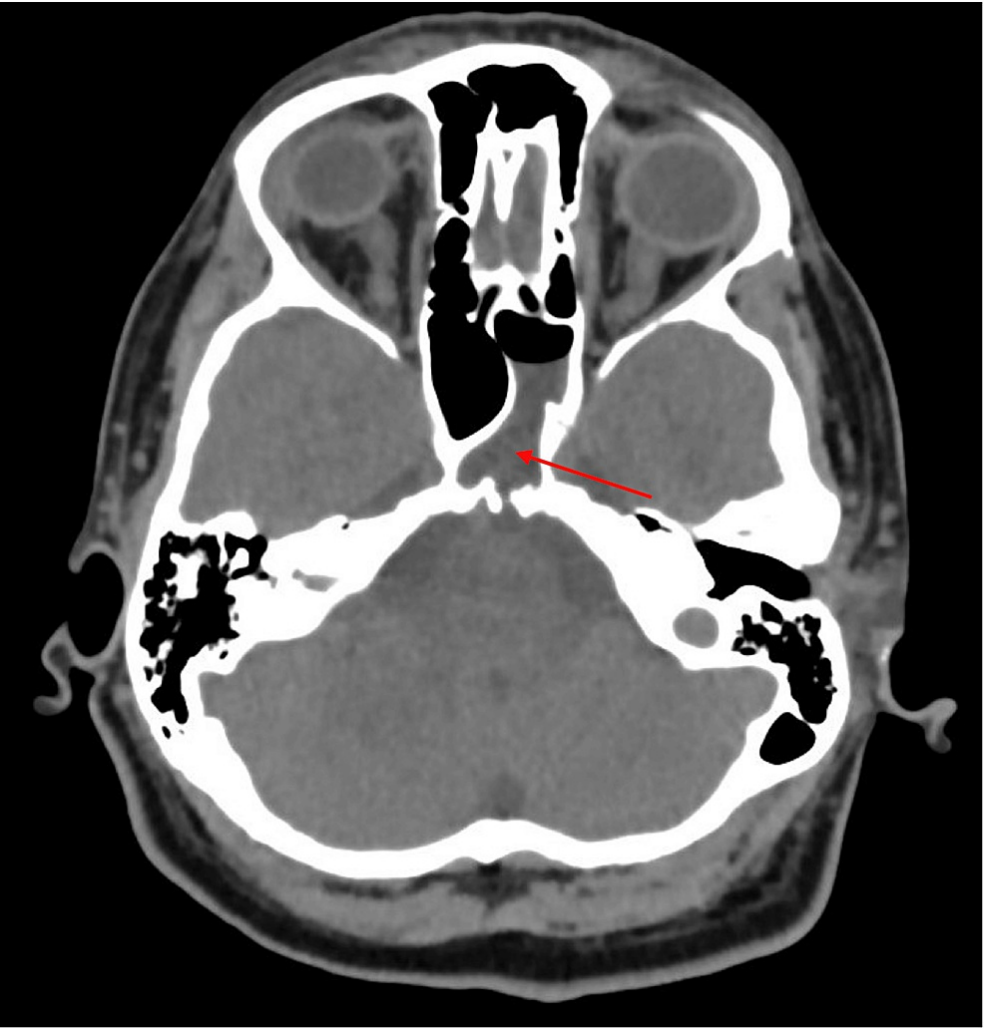
CT intracranial angiogram at hospital admission The red arrow highlights sphenoid sinus contrast extravasation concerning for a CSF leak.

**Figure 2:**
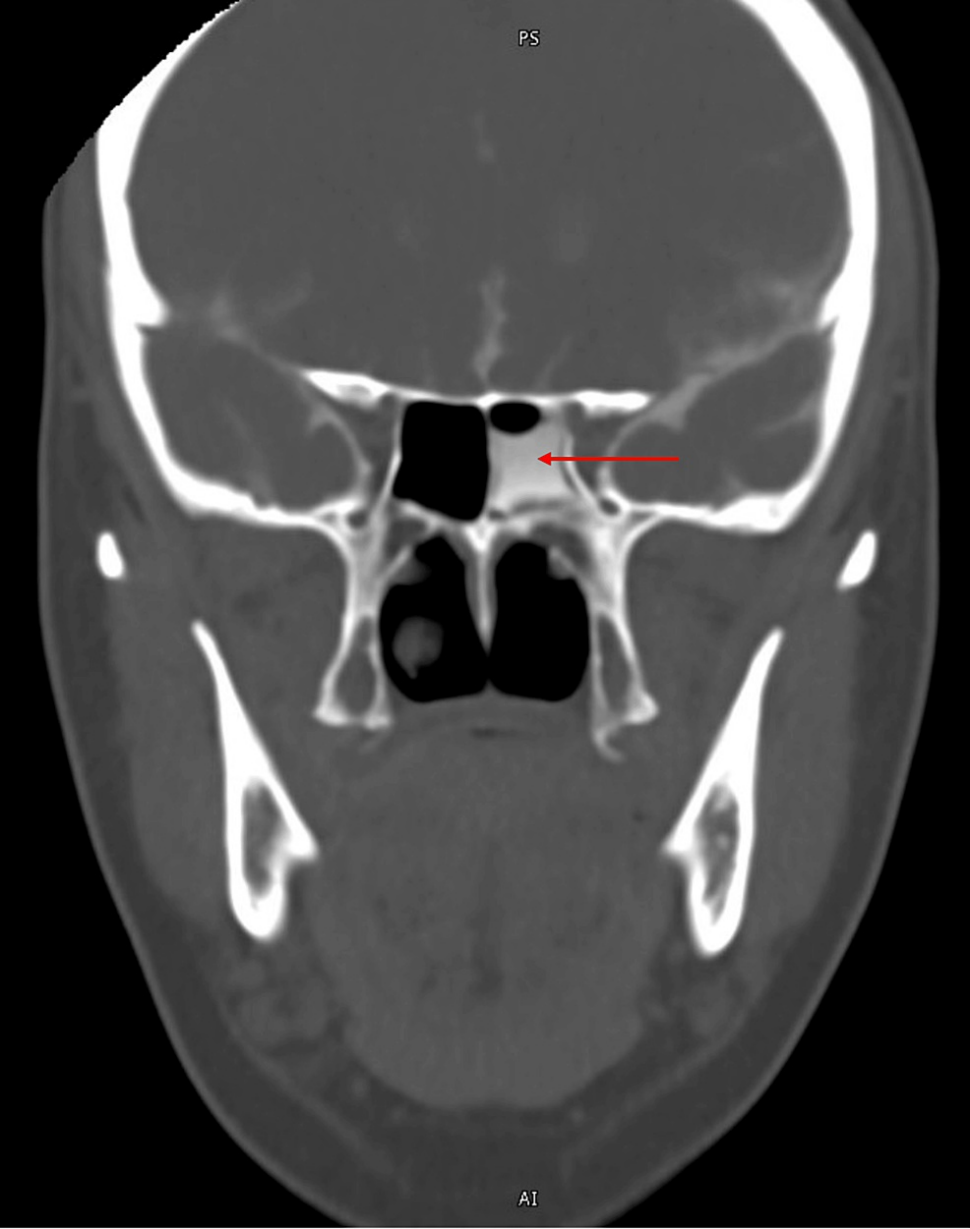
CT cisternogram performed due to a high suspicion of a CSF leak in the CT sinus The red arrow highlights contrast extravasation through a bony defect of the posterior sphenoid sinus.

The patient is a 37-year-old female with no significant past medical history who presented to the emergency department (ED) with endorsements of a headache that started two days prior with associated nausea, headache, and blurry vision. She described her headache as "sharp and stabbing" with radiation down her neck with associated neck pain and stiffness. She rated her headache a 10/10 and denied fever and chills but noted a “clear, salty fluid draining from her left nares” over the past several weeks. Vitals at presentation included a heart rate of 118 beats per minute; otherwise, they were stable. The patient presented to her primary care physician earlier in the week and was prescribed Flonase without relief. Upon admission, computed tomography (CT) head was performed and revealed concerns for acute sinusitis. CT angiography of the intracranial and extracranial vasculature was performed and was non-revealing for an acute intracranial abnormality. Complete blood count (CBC) was remarkable for a white blood cell (WBC) count of 27.2 x10^3/uL and C-reactive protein (CRP) of 173.3 mg/L. A respiratory virus panel was performed and was unremarkable for COVID, influenza, and respiratory syncytial virus. Due to concerns about meningitis, empiric antibiotic therapy, including vancomycin and ceftriaxone, were initiated. Compazine, Benadryl, and IV fluids were administered without relief. Initially, the patient deferred a lumbar puncture given a negative experience in the past. She was admitted to the medical service for further management. Upon admission, the patient was agreeable to undergo a diagnostic lumbar puncture. A 22-gauge lumbar puncture needle was advanced with multiple attempts to access the spinal canal without success. Interventional Radiology was consulted for lumbar puncture, which revealed 54 mg/dL protein, 70 mg/dL glucose, 875 nucleated cells, and 435 red blood cells. Meningitis and encephalitis panel, CSF herpes simplex virus (HSV)-1, and cryptococcal antigen were negative. CSF gram culture and stain revealed no organismal growth; however, blood cultures were positive for gram-positive cocci in pairs and chains, ultimately revealing salivarius group viridans streptococcus. Infectious Disease was consulted for further antibiotic guidance and ultimately recommended continuing IV vancomycin with an area under the curve (AUC) of 400-600, IV ceftriaxone 2g Q12H with the addition of oral metronidazole 500 mg t.i.d. A transthoracic echocardiogram (TTE) was also recommended per Infectious Disease, which revealed an ejection fraction of 63.4% without evidence of visible vegetation. One day after admission, a revision was made to the initial CT intracranial angiogram concerning for dehiscence of the left posterior of the sphenoid sinus with a CSF density-like material and air-fluid level (Figure 1). These findings, in combination with a positive beta-2 transferrin sampled from the patient’s nasal discharge, raised suspicion of a CSF leak. Further evaluation with CT cisternogram revealed extravasation of contrast through the bony defect involving the posterior wall of the left sphenoid sinus with contrast filling the sphenoid sinus and extending into the posterior sinus drainage pathway (Figure 2). Otolaryngology was consulted and the patient ultimately underwent bilateral wide sphenoidotomies and posterior septectomy along with repair of the CSF leak using an abdominal fat graft and left nasoseptal flap. The procedure was tolerated without complications and her symptoms continued to improve. A magnetic resonance imaging (MRI) brain was performed to rule out an encephalocele or intracranial tumor per Otolaryngology, which was unremarkable. Prior to discharge, the patient's IV vancomycin was discontinued, and she was continued on IV ceftriaxone 2 g Q 24 hours and oral metronidazole 500 mg t.i.d. for a two-week therapy duration and discharged. One week post-discharge, the patient presented again to an outside ED due to concerns about seizure-like activity. While receiving medications through IV push, the patient began shaking all over and her pupils became dilated. This episode lasted minutes and resolved on its own with endorsements of fatigue thereafter. A leukocytosis of 17.1x10^3/uL was noted on presentation. She declined to undergo further imaging of her brain due to recent imaging during prior hospitalization and was ultimately transferred to our facility for further evaluation by otolaryngology in the setting of the recent CSF leak repair. Per Otolaryngology, a CT brain with and without contrast revealed interval postoperative changes from bilateral sphenoidotomies and posterior septectomy with the repair of a previously identified CSF leak. Neurology was also consulted during the admission due to concerns about seizure-like activity. The patient underwent a continuous video EEG, which was unrevealing for abnormal epileptiform discharges. Per further assessment, it was believed the patient's initial concern for seizure-like activity was likely related to vasovagal syncope in his sitting of pushing the patient's antibiotics too quickly. Because of this, the patient's outpatient IV ceftriaxone was adjusted to include an infusion over 30 minutes rather than a push dose. The patient was ultimately discharged. She followed up with Otolaryngology two weeks after discharge and was found to be doing well without headache, nausea, vomiting, blurred vision, or clear nasal drainage concerning for a CSF leak. 

## Discussion

Spontaneous CSF leaks are rare, accounting for roughly 3-5% of all CSF leaks [12]. As CSF leaks present with non-specific symptoms, including headache, nausea, and neck pain, specific patient demographics can be examined to determine the likelihood of developing a specific type of CSF leak [1]. Mamlouk et al. explored patient demographics via a retrospective review of 65 patients diagnosed with CSF leak on CT myelography, including patient age, body mass, and anatomical level of the CSF leak [13]. Patients of increasing age were more likely to have a type 3 CSF leak secondary to a venous fistula, whereas those with a higher BMI were more likely to have a type 2 CSF leak [13]. Stratification by patient characteristic can be helpful; however, in cases where the leak is idiopathic secondary to a bony defect, such as in our patient with a noted sphenoid sinus defect, stratification and diagnosis can be much more difficult [14]. Defects associated with idiopathic CSF leaks include the cribriform plate, sphenoid bone, temporal bone, fovea ethmoidal, and frontal bone [14]. Compared to other locations, a spontaneous CSF leak attributed to the sphenoid sinus, as seen in our patient, is quite rare, as only 7% from this location are known to be spontaneous [14].

When one presents with symptoms concerning for a CSF leak, several steps must be taken to establish a diagnosis. If clear rhinorrhea is present on the exam, the sample can be tested for beta-2 transferrin, a glycoprotein found in CSF [15]. Testing this protein has a sensitivity of 97% and specificity of 99% [15]. Testing of our patient’s rhinorrhea tested positive for beta-2 transferrin, making the diagnosis of a CSF leak highly likely. Even in cases where rhinorrhea is positive for beta-2 transferrin, radiographic imaging should be performed to determine the etiology of the CSF leak [16]. In cases where beta-2 transferrin is negative, radiographic imaging is essential [16]. When a CSF leak is suspected, a CT skull and CT sinus can be performed. If negative, an MRI can be performed. When both imaging modalities are utilized, the sensitivity and specificity increase to 95% and 100% for the diagnosis of a CSF leak [16-18]. In our case, CT intracranial and extracranial were initially performed due to endorsements of neck stiffness on admission. After initially reading as unremarkable, a revision was made to the initial read concerning for dehiscence of the sphenoid sinus with an associated CSF density. In the setting of spontaneous CSF leaks, MRI may further characterize the etiology including evidence of a meningocele [15]. Idiopathic intracranial hypertension may present on MRI with parenchymal thickening and intracranial tumors may also be visualized [1]. In our case, the MRI was negative for an encephalocele or acute intracranial process, instead identifying a posterior defect of the sphenoid sinus. If initial CT and MRI are non-revealing, CT cisternography can be performed, which is the gold standard for the diagnosis of a CSF leak [18]. In our case, the CT cisternogram revealed contrast extravasation through a bony defect localized to the sphenoid sinus (Figure 2).

CSF leaks are not without complications and management is case-specific. In patients presenting with signs of infection, lumbar puncture should be performed, as this defect increases the risk for meningitis, especially in those with a traumatic etiology leading to a CSF leak [1]. In our case, lumbar puncture was non-revealing for infection. Management of a CSF leak is dependent upon multiple factors, including the size and etiology of the leak. Smaller leaks may resolve on their own and can be managed with supportive therapy, including hydration, bed rest, and caffeine avoidance, whereas large leaks often need surgical repair [1]. In patients with an anterior skull base defect, endoscopic repair is preferred due to the high first-procedure success rate of 86-100% [19]. In patients with a temporal skull defect, a more invasive mastoidectomy or craniotomy may be preferred [20]. Due to the location of the sphenoid sinus, our patient underwent a successful endoscopic sphenoidotomy and posterior septectomy.

## Conclusions

A CSF leak is a rare, life-threatening pathology that can present with non-specific symptoms, including headache, nausea, and neck pain. Etiologies include trauma, recent surgery, intracranial hypertension, intracranial tumors, and infrequent, idiopathic causes leading to a spontaneous CSF leak. Here, we present a case presenting with non-specific symptoms, including headache and neck pain, diagnosed with a spontaneous leak and treated appropriately. By presenting this case, we aim to raise awareness to prevent a delay in diagnosis and highlight features of this rare pathology.
